# Awareness and Incorporation of the Dental Home Concept Among General Dentists and Dental Therapists in Malaysia

**DOI:** 10.7759/cureus.57421

**Published:** 2024-04-01

**Authors:** Munalizaini Mukhtar, Norkhafizah Saddki, Zuliani Mahmood

**Affiliations:** 1 Dental Public Health Unit, School of Dental Sciences, Universiti Sains Malaysia, Health Campus, Kelantan, MYS; 2 Pediatric Dentistry Unit, School of Dental Sciences, Universiti Sains Malaysia, Health Campus, Kelantan, MYS

**Keywords:** dental home, dental nurses, general dentists, child oral health, dental care

## Abstract

Background

The dental home concept (DHC) refers to an approach in oral healthcare that emphasizes establishing a long-term, comprehensive, and family-centered relationship between a patient and their primary dental care. This study determined the awareness and incorporation of the DHC among general dentists and dental therapists in Malaysia.

Methodology

A total of 154 general dentists and 137 dental therapists providing oral healthcare services at the Ministry of Health (MOH) primary care facilities throughout Malaysia participated in this cross-sectional study. A self-administered questionnaire was used to measure the respondents’ awareness of the DHC and incorporation of the DHC characteristics into their practice.

Results

Most dentists and dental therapists (61.7% and 67.2%, respectively) had not heard of the term DHC and were unaware of the concept. The respondents’ awareness was not associated with their age, sex, years of service, facility location, and percentage of treatment given to children aged five years and below. However, most dentists and dental therapists responded positively about incorporating most DHC characteristics into their current practice.

Conclusions

Most dentists and dental therapists serving the MOH primary oral healthcare facilities were unaware of the DHC, although most DHC characteristics have already been incorporated into their practice. This study provides evidence of the incorporation of the DHC into the MOH primary oral healthcare services and suggests an effort to increase the awareness of the workforce regarding the concept and its implementation.

## Introduction

Dental caries has affected more than 530 million children worldwide [1]. With an estimated prevalence of 48% among preschool children, early childhood caries is a major public health challenge [2]. Despite the reported declining prevalence in many countries, the global burden of dental caries has remained relatively unchanged over the past 30 years [3]. It does not only place a significant burden and cost on society but can also negatively affect the quality of life of the child and the family members [4-6].

In 2001, the American Academy of Pediatric Dentistry (AAPD) introduced the dental home concept (DHC) to help children and their families institute a lifetime of good oral health [7]. Adopted from the concept of medical home by the American Academy of Pediatrics, the dental home was defined as “the ongoing relationship between the dentist and the patient, inclusive of all aspects of oral health care delivered in a comprehensive, continuously accessible, coordinated, and family-centered way,” which should be established before the age of 12 months [8]. Early professional dental care includes anticipatory guidance for parents and periodic follow-up visits based on the child’s risk of oral diseases [9]. By providing preventative, emergency, and holistic oral health services, as well as referral to dental professionals as necessary [8], the DHC has been shown to be effective in reducing the prevalence and risk of early childhood caries [10,11].

In Malaysia, the Early Childhood Oral Health Care Program was introduced by the Ministry of Health (MOH) in 2008 to promote and maintain good oral health of toddlers toward achieving their optimum growth and development. In accordance with the DHC, the program recommends oral examination and risk assessment of children to be done within six months of eruption of the first tooth or at the age of one year. Aiming to provide appropriate parental guidance, the program targets postnatal mothers and parents/carers of children aged four years and below who are seen at the MOH maternal and child health clinics and community health clinics for health services such as immunization, childcare centers established by government bodies, and registered privately owned childcare facilities within the constraints of local resources [12]. However, after 10 years, the implementation of the program did not seem to achieve a satisfactory outcome. In 2018, only 18.3% of toddlers attended primary oral healthcare services [13]. Additionally, although there has been a decline in caries prevalence in five-year-old children from 76.2% in 2005 to 71.3% in 2015, no significant reduction in caries severity was observed with a mean number of decayed and filled teeth of 4.83 in 2015 compared to 5.50 in 2005 [14].

By definition, a dental home is described as a continuing relationship between the dentist and the patient [8]. In Malaysia, in addition to general dentists or dental officers (DOs), dental therapists (DTs) play a tremendous role in providing preventive and limited restorative dental care for children and young adults under the age of 18 years [15]. Notwithstanding the existing guidelines on oral healthcare for toddlers and preschool children [12,16,17], the extent to which the MOH DOs and DTs are aware of the DHC is not known. Hence, this study aimed to assess the awareness and incorporation of the DHC among the MOH DOs and DTs.

## Materials and methods

Study design and population

This was a cross-sectional study of the MOH DOs and DTs serving the primary oral healthcare facilities in Malaysia. Dental specialists serving at primary oral healthcare facilities, DOs who were attached to dental specialists or were based at administrative dental offices, and DTs who held administrative positions were excluded.

The largest affordable sample size was obtained from the objective of determining the DHC incorporation among DOs in Malaysia using the formula to estimate a single proportion with a 95% confidence interval (CI). The proportion was estimated at 21% based on the findings by Hammersmith et al. [18] among general dentists in Ohio. At a precision of 0.05, a sample size of 255 was calculated. Anticipating a 40% non-response rate, a sample size of 360 was decided. Ethical approval for this study was obtained from the Universiti Sains Malaysia Human Research and Ethics Committee (USM/JEPeM/21010037) and the MOH Medical Research and Ethics Committee (NMRR-21-214-57945 (IIR)).

A multistage sampling method was used to obtain a sample of 360 MOH DOs and DTs from 569 primary oral healthcare facilities in 13 states and three federal territories of Malaysia. Of the 569 facilities, 236 were in urban areas and 333 were in rural areas. Simple random sampling was used to choose 180 urban and 180 rural facilities from their respective stratum. Another simple random sampling was applied to select 90 eligible DOs and 90 eligible DTs from their respective facility, which means either one DO or one DT was selected from each facility.

Research tool

A self-administered questionnaire adapted from Hammersmith et al. [18] was used to assess the awareness and incorporation of DHC among the respondents. Three items assess the respondents’ awareness of the DHC. The first item asks if the respondents are aware or had ever heard of the term DHC, the second item is on the respondents’ understanding of dental home, and the third item asks when a dental home should be established.

The incorporation of DHC for children aged five years and below has 39 items. The items were arranged into seven dental home characteristics or domains as follows: accessible (nine items), compassionate (four items), family-centered (four items), comprehensive (10 items), culturally effective (five items), coordinated (four items), and continuous (three items). The following five response options were given: 1 for “never,” 2 for “hardly ever,” 3 for “occasionally,” 4 for “fairly often,” and 5 for “very often.” Responses “very often,” “fairly often,” “occasionally,” and “hardly ever” were grouped to indicate incorporation of DHC (positive response) while the “never” response was interpreted as the absence of DHC incorporation in the current practice (negative response).

The original English version of the questionnaire underwent a translation and adaptation process recommended by Beaton et al. [19]. The content validity of the Malay-translated version was established by a panel of experts consisting of one Dental Public Health Specialist, one Pediatric Dentist, and one MOH DO. The validated questionnaire was converted into an online form using the Google Forms software. Questions on sociodemographic background, namely, age, sex, year of graduation, years of service, and percentage of treatment given to children aged five and below were also included. A pilot testing was done using the online version of the questionnaire on six DOs and five DTs from two primary oral healthcare facilities not selected for the study.

Data collection

Selected eligible DOs and DTs were contacted individually via their official email and invited to participate in the study. In addition to sociodemographic questions and items that assessed awareness and incorporation of DHC, the online survey form also included a participant information sheet that had information about the study objectives, eligibility criteria, procedures, and other essential information about the study, as well as an informed consent statement that asked if they agree to participate by answering “yes” or “no.” A “yes” answer indicated their consent to participate in the study, and they were requested to proceed to the next page to start answering the questionnaire.

The respondents were requested to email the main author if they had any difficulty comprehending any of the items. The response rate was monitored closely. After one week, a reminder email was sent to the non-responders, followed by the second and the third (final) reminder at a one-week interval.

Statistical analysis

Data generated by the Google Spreadsheet was downloaded and transferred into the SPSS Software, version 24.0 (IBM Corp., Armonk, NY, USA) for statistical analysis. Descriptive statistical analysis was performed to obtain the frequency and percentage of categorical variables and the mean and standard deviation (SD) of numerical variables. Additionally, logistic regression analysis was conducted to determine the association between the respondents’ awareness of DHC and the following factors: age, sex, job/post, years of service, location of primary oral healthcare facilities, and percentage of treatment given to children aged five and below. The level of significance for this analysis was set at a p-value of less than 0.05.

## Results

Sociodemographic characteristics

A total of 291 respondents completed the questionnaire, giving a response rate of 80.3%. The sociodemographic background of respondents is presented in Table 1. Most DOs, 136 (88.3%), and all 137 (100.0%) DTs were female. The mean age of the DOs was 30.3 years (SD = 0.33) and the DTs was 35.5 years (SD = 0.63). While about half of the DTs were 35 years and older, most DOs, 134 (87.0%), were mainly under the age of 35. Consistent with the age profile, the mean duration of service in the MOH was also longer for DTs at 11.8 years (SD = 0.60) compared with DOs at 5.0 years (SD = 0.31). Most DTs, 105 (76.7%), had been in the service for more than five years, whereas most DOs, 99 (64.3%), had no more than five years of work experience. The mean percentage of treatment given to children aged five and below was higher in DTs at 56.2% (SD = 2.11) than in DOs at 27.3% (SD = 1.13).

**Table 1:** Profile of the respondents (n = 291). DO = dental officers; DT = dental therapists

Variable	Frequency (%)	
	DO (n = 154)	DT (n = 137)
Age group (year)		
<30	80 (51.9)	45 (32.8)
30–34	54 (35.1)	19 (13.9)
35–39	16 (10.4)	27 (19.7)
40–44	2 (1.3)	31 (22.6)
45–49	2 (1.3)	11 (8.0)
≥50	0 (0.0)	4 (2.9)
Sex		
Male	18 (11.7)	0 (0.0)
Female	136 (88.3)	137(100.0)
Years of service		
≤5	99 (64.3)	32 (23.3)
6–10	42 (27.3)	27 (19.7)
11–15	10 (6.5)	30 (21.9)
16–20	3 (1.9)	33 (24.1)
21–25	0 (0.0)	12 (8.8)
>25	0 (0.0)	3 (2.2)
Location of facility		
Rural	64 (41.6)	47 (34.3)
Urban	90 (58.4)	90 (65.7)
Percentage of treatment given to children aged five and below		
≤25	73 (47.4)	7 (5.1)
26–50	77 (50.0)	72 (52.6)
51–75	4 (2.6)	21 (15.3)
>75	0 (0.0)	37 (27.0)

Awareness of the dental home concept

Table 2 shows the awareness of the DHC among the MOH DOs and DTs. Most MOH DOs, 95 (61.7%), and 92 (67.2%) DTs had not heard of the DHC. Only around a quarter of the DOs, 41 (26.6%), and less than one-fifth of the DTs, 24 (17.5%), knew that DHC is an ongoing relationship between a dentist and the patient. Additionally, less than half of the DOs, 56 (36.4%), and 54 (39.4%) DTs knew that the DHC should be established no later than one year of age. None of the investigated respondents’ profiles was found to be associated with their awareness of the DHC (Table 3).

**Table 2:** Awareness of the dental home concept among respondents (n = 291). DO = dental officers; DT = dental therapists

Variable	Frequency (%)	
	DO (n = 154)	DT (n = 137)
Have heard of the dental home concept		
No	95 (61.7)	92 (67.2)
Yes	59 (38.3)	45 (32.8)
The dental home is described by the ongoing relationship between whom		
I don’t know	21 (13.6)	43 (31.4)
A dentist and the patient	41 (26.6)	24 (17.5)
Any medical or dental professional and the patient	92 (59.7)	70 (51.1)
By when should the dental home be established		
I don’t know	58 (37.7)	57 (41.6)
No later than one year of age	56 (36.4)	54 (39.4)
No later than three years of age	40 (26.0)	26 (19.0)

**Table 3:** Simple logistic regression analysis of respondents’ awareness of the dental home concept (n = 291). Simple logistic regression analysis was used as appropriate. The level of significance for this analysis was p < 0.05. ^a^: Likelihood ratio (LR) test. DO = dental officers; DT = dental therapists; df = degree of freedom

Variable	Crude odds ratio	95% confidence interval	Chi-square statistics (df)^a^	P-value^a^
Age (year)				
<35	1.00			
≥35	1.58	0.95, 2.62	3.108 (1)	0.078
Sex				
Male	1.00			
Female	0.53	0.21, 1.39	1.633 (1)	0.201
Job/Post				
DT	1.00			
DO	1.27	0.78, 2.06	0.945 (1)	0.331
Years of service				
≤5	1.00			
>5	0.99	0.61, 1.60	0.002 (1)	0.964
Location of facility				
Rural	1.00			
Urban	1.19	0.72, 1.95	0.454 (1)	0.500
Percentage of treatment given to children aged five and below				
≤50	1.00			
>50	0.75	0.41, 1.37	0.906 (1)	0.341

Incorporation of the dental home concept

Table 4 shows responses from the MOH DOs and DTs regarding the incorporation of the DHC characteristics into their oral healthcare practice. Most DOs and DTs responded positively to all characteristics related to the incorporation of accessible, compassionate, family-centered, culturally effective, coordinated, and continuous services into the practice. Only one item in the comprehensive domain (the clinic provides special equipment and appliance therapy) had low positive responses from both DOs and DTs at 47 (30.5%) and 71 (51.8%), respectively.

**Table 4:** Respondents incorporating the dental home concept into their practice (n = 291). DO = dental officers; DT = dental therapists

Variable	Frequency (%)	
	DO (n = 154)	DT (n = 137)
Accessible		
New patients are scheduled for examination within four weeks	135 (87.7)	121 (88.3)
Established patients are scheduled for treatment within four weeks	146 (94.8)	124 (90.5)
Patients are seated in the treatment room within a reasonable amount of time of their scheduled appointment time	153 (99.4)	134 (97.8)
The clinic is easily accessible via public transport	137 (89.0)	123 (89.8)
Emergency care is provided during office hours	148 (96.1)	134 (97.8)
Emergency care is provided after hours at the nearest hospital	139 (90.3)	113 (82.5)
The clinic makes it easy for patients to schedule appointments	149 (96.8)	135 (98.5)
The clinic accepts supporting documents for fee exemption	152 (98.7)	136 (99.3)
Services are provided for patients with special needs	152 (98.7)	132 (96.4)
Compassionate		
Compassionate staff who recognize socioeconomic issues	154 (100.0)	136 (99.3)
The staff attempt to communicate effectively with patients with low literacy	154 (100.0)	135 (98.5)
The clinic offers less expensive, clinically acceptable alternative treatment plans for patients who cannot afford optimal care	151 (98.1)	130 (94.9)
The clinic will arrange payment plans	129 (83.8)	104 (75.9)
Family-centered		
The clinic provides oral health recommendations specific and individualized to each family	152 (98.7)	135 (98.5)
Dental Officers and Dental Therapists involve parents in making treatment decisions and when providing oral hygiene instructions	154 (100.0)	137 (100.0)
Dental Officers and Dental Therapists consult parents about different behavior management techniques that can be used	154 (100.0)	134 (97.8)
The clinic encourages and evaluates parental compliance for home care	154 (100.0)	136 (99.3)
Comprehensive		
Provides services for children aged zero to two years, including examination, prophylaxis, restorative treatment, and extraction	152 (98.7)	125 (91.2)
Provides services for children aged three to five years, including examination, prophylaxis, restorative treatment, and extraction	154 (100.0)	137 (100.0)
Provides dietary counseling services and anticipatory guidance for children	151 (98.1)	136 (99.3)
Provides caries risk assessment for children according to the protocol	154 (100.0)	137 (100.0)
The clinic encourages emergency patients to return for comprehensive care	154 (100.0)	135 (98.5)
Dental Officers make referrals to Medical Officers, as necessary	151 (98.1)	133 (97.1)
Dental Officers make referrals to dental specialists, as necessary	154 (100.0)	137 (100.0)
Dental Officers write prescriptions, as necessary	154 (100.0)	136 (99.3)
The clinic provides special equipment and appliance therapy	47 (30.5)	71 (51.8)
Dental Officers provide range of services for behavior management	149 (96.8)	118 (86.1)
Culturally effective		
The clinic tries to overcome the communication gap with patients from different sociocultural backgrounds	154 (100.0)	133 (97.1)
The clinic treats patients with sensory impairments	152 (98.7)	129 (94.2)
The clinic has staff, decor, procedures, and policies that are appropriate for patients from different sociocultural backgrounds	142 (92.2)	121 (88.3)
Recognize culturally specific treatment needs and priorities of the local ethnic groups	151 (98.1)	124 (90.5)
The clinic provides oral health information printed in Malay and other languages	144 (93.5)	127 (92.7)
Coordinated		
The clinic coordinates care for patients requiring complex dental treatment	131 (85.1)	122 (89.1)
The clinic coordinates care for patients requiring complex medical treatment	126 (81.8)	113 (82.5)
The clinic coordinates care with the Medical Officers	151 (98.1)	132 (96.4)
The clinic coordinates care with social services, schools, and care facilities	149 (96.8)	136 (99.3)
Continuous		
The clinic provides recall visits and continuing care over the long term	154 (100.0)	137 (100.0)
Dental Officers and Dental Therapists schedule patients for recall	153 (99.4)	136 (99.3)
The clinic participates in community programs	153 (99.4)	136 (99.3)

## Discussion

Integrating dental home with existing MOH programs, particularly the Early Childhood Oral Health Care Program and the Antenatal Mothers Program, will enable seamless care which can contribute to the prevention and control of early childhood caries among Malaysian children. The establishment of the dental home has validity and the potential to enhance the nation’s oral health, beginning in early childhood. Some dental home qualities may have already been practiced in the MOH oral health services, but perhaps not all the characteristics are fully integrated. This study was conducted to evaluate the awareness of the DHC among the DOs and DTs working at the MOH primary healthcare facilities and the incorporation of the DHC characteristics into their practice.

Our study showed that most MOH DOs and DTs had not heard of the term DHC and could not correctly identify the two aspects of the DHC; that it is an ongoing relationship between a dentist and the patient, and it should begin no later than one year of age. A study by Hammersmith et al. [18] in Ohio also showed that most general dental practitioners were not aware of the term (82%) and could not correctly identify both DHC features (93%), as opposed to pediatric dentists who were mostly familiar with the term (78%) and were correct about the features (59%), possibly attributed to their affiliation with the AAPD. A qualitative study by VanMalsen et al. [20] among dentists and dental hygienists in Alberta, Canada also demonstrated that leadership and support from regulatory bodies and professional associations had a positive influence on the practitioners’ provision of infant-toddler dental homes.

All DTs in this study were female because DT training for public services is limited to females [21]. Most DOs were also female, reflecting the higher number of female students in most dental faculties in Malaysia [22]. The percentage of treatment for five-year-olds was higher in the DT group, in line with the main job description of DTs, who are at the forefront of providing oral healthcare for children in Malaysia [15], complementing the role of DOs in meeting the oral health needs of the young generation. The awareness of the DHC among DOs and DTs in this study was not associated with their age, sex, years of service, facility location, percentage of treatment given to children aged five years and below, and their being DOs or DTs. Hammersmith et al. [18], however, showed that, in addition to being a specialist in pediatric dentistry, dentists who graduated recently were more likely to be aware of the DHC. Most DOs in this study were recent graduates who had worked for not more than five years, yet were unaware of the concept.

Nowak and Casamassimo [9] delineated seven DHC characteristics, namely, accessible, compassionate, family-centered, comprehensive, culturally competent, coordinated, and continuous. Although most MOH DOs and DTs in this study were unaware of the DHC term and concept, it is good to note that almost all DHC characteristics have already been incorporated into their practice. These favorable findings can be attributed to the strong commitment of the MOH to promoting and maintaining the good oral health of toddlers and preschool children through the development of guidelines, programs, and standard operating procedures that are mainly consistent with the DHC characteristics [12,16,17,23,24]. Nevertheless, incorporating dental home into practice without knowing or understanding the concept and its characteristics can have a negative impact on the quality and sustainability of the service. Hence, efforts should be made by the MOH to increase awareness of the DOs and DTs regarding the DHC and its characteristics, and how the characteristics can be and have been incorporated into oral health practice and services.

The DHC characteristic with the lowest percent score among both DOs and DTs in this study was providing specific equipment and appliance therapy which are mainly used in procedures to modify or guide the children’s dentofacial development and growth. In the MOH, the oral healthcare program is divided into four main services, namely, oral health promotion, primary oral healthcare, specialist oral healthcare, and community oral healthcare. While the dental home is established under the primary oral healthcare service, the provision of specific equipment and appliance therapy is provided mainly under the specialist oral healthcare service by pediatric dentists and orthodontists. In the public sector, pediatric dentistry is a hospital-based specialization, whereas orthodontics is a non-hospital-based specialization provided at primary care facilities. Orthodontic treatment is currently offered by only about one-tenth of all primary care facilities in Malaysia. The unavailability of pediatric dentists and the shortage of orthodontists at primary oral healthcare facilities could explain the low incorporation of specific equipment and appliance therapy for children aged five years and below.

The key strength of this study was the study population comprised of DOs and DTs who were sampled from urban and rural primary oral healthcare facilities from all states and federal territories of Malaysia. Hence, allowing the generalization of the findings to all MOH DOs and DTs in the country. However, our study has a limitation relating to the potential bias from the use of a self-administered questionnaire. The tendency of the respondents to provide acceptable or desirable answers might have inflated the positive responses, resulting in an inaccurate reflection of their awareness and incorporation of the DHC into the practice. Our results, therefore, need to be interpreted with caution.

## Conclusions

The MOH Malaysia is committed to improving the oral health status of Malaysians, which can be achieved through the establishment of the DHC that emphasizes early contact between a child and the oral healthcare provider, followed by delivery of accessible, compassionate, family-centered, comprehensive, culturally effective, coordinated, and continuous oral healthcare. However, the findings of our study showed that most MOH DOs and DTs had not heard of the term DHC and were unaware of its concept, although most DHC characteristics have already been incorporated into their oral healthcare practice. To ensure the sustainability and continual quality of the services, efforts should be made by the MOH to increase the DO’s and DT’s awareness of the DHC and its characteristics. Another way to improve awareness of the DHC among the dental workforce is by integrating this element into the undergraduate dental degree curriculum and dental therapy diploma curriculum. By incorporating this element into the curriculum, the new generation of DOs and DTs would be better equipped to help ensure the services provided reach the benefits of a dental home.

## Appendices

**Table 5:** Questionnaire assessing the awareness and incorporation of the dental home concept among respondents.

Part A: Socio-demographic Background							
Age			......... years				
Sex			( ) Male ( ) Female				
In what year did you receive your dental doctor degree or dental therapist diploma?			Year .................				
You have served the Ministry of Health for:			......... years				
Are you a:			( ) Dental Officer ( ) Dental Therapist				
What percentage of your treatment is given to children ages 5 and under?			........... %				
Part B: Incorporation of Dental Home Concept in the Ministry of Health Oral Health Care Services							
Which of the following applies to your oral health care practice for children ages 5 and under?							
		Never		Seldom	Sometimes	Often	Very often
1	New patients are scheduled for examination within four weeks	( )		( )	( )	( )	( )
2	Established patients are scheduled for treatment within four weeks	( )		( )	( )	( )	( )
3	Patients are seated in the treatment room within a reasonable amount of time (on average 15 minutes) of their scheduled appointment time	( )		( )	( )	( )	( )
4	The clinic is easily accessible via public transport	( )		( )	( )	( )	( )
5	Emergency care is provided during office hours	( )		( )	( )	( )	( )
6	Emergency care is provided after hours at the nearest hospital	( )		( )	( )	( )	( )
7	The clinic makes it easy for patients to schedule appointments (for example, by means of telephone or Internet)	( )		( )	( )	( )	( )
8	The clinic accepts supporting documents for fee exemption	( )		( )	( )	( )	( )
9	Care is provided for patients with special needs (for example, Down syndrome, cerebral palsy, mental retardation or developmental delay)	( )		( )	( )	( )	( )
Which of the following applies to your oral health care practice for children ages 5 and under?							
		Never		Seldom	Sometimes	Often	Very often
1	Compassionate staff who recognize socioeconomic issues	( )		( )	( )	( )	( )
2	The staff attempts to communicate effectively with patients with low literacy	( )		( )	( )	( )	( )
3	The clinic offers less expensive, clinically acceptable alternative treatment plans for patients who cannot afford optimal care (for example, extraction versus pulp therapy and restoration)	( )		( )	( )	( )	( )
4	The clinic will arrange payment plans (for example, fee reduction and incremental payment)	( )		( )	( )	( )	( )
Which of the following applies to your oral health care practice for children ages 5 and under?							
		Never		Seldom	Sometimes	Often	Very often
1	The clinic provides oral health recommendations specific and individualized to each family	( )		( )	( )	( )	( )
2	Dental Officers and Dental Therapists involve parents in making treatment decisions and when providing oral hygiene instructions	( )		( )	( )	( )	( )
3	Dental Officers and Dental Therapists consult parents about different behavior management techniques that can be used	( )		( )	( )	( )	( )
4	The clinic encourages and evaluates parental compliance with home care	( )		( )	( )	( )	( )
Which of the following applies to your oral health care practice for children ages 5 and under?							
		Never		Seldom	Sometimes	Often	Very often
1	Provides care for children aged 0 to 2 years, including examinations, prophylaxis, restorative treatment, and extractions	( )		( )	( )	( )	( )
2	Provides care for children aged 3 to 5 years, including examinations, prophylaxis, restorative treatment, and extractions	( )		( )	( )	( )	( )
3	Provides dietary counseling and anticipatory guidance (age-appropriate counseling) for children	( )		( )	( )	( )	( )
4	Provides caries risk assessment for children according to the protocol	( )		( )	( )	( )	( )
5	The clinic encourages emergency patients to return for comprehensive care	( )		( )	( )	( )	( )
6	Dental Officers make referrals to Medical Officers, as necessary	( )		( )	( )	( )	( )
7	Dental Officers make referrals to dental specialists, as necessary	( )		( )	( )	( )	( )
8	Dental Officers write prescriptions, as necessary	( )		( )	( )	( )	( )
9	The clinic provides special equipment and appliance therapy (for example, space maintainers and habit appliances)	( )		( )	( )	( )	( )
10	Dental Officers provide a range of services for behavior management	( )		( )	( )	( )	( )
Which of the following applies to your oral health care practice for children ages 5 and under?							
		Never		Seldom	Sometimes	Often	Very often
1	The clinic tries to overcome the communication gap with patients from different sociocultural backgrounds	( )		( )	( )	( )	( )
2	The clinic treats patients with sensory impairments (for example, visual or hearing)	( )		( )	( )	( )	( )
3	The clinic has staff, decor, procedures, and policies that are appropriate for patients from different sociocultural backgrounds	( )		( )	( )	( )	( )
4	Recognize culturally specific treatment needs and priorities of the local ethnic groups	( )		( )	( )	( )	( )
5	The clinic provides oral health information printed in Malay and other languages	( )		( )	( )	( )	( )
Which of the following applies to your oral health care practice for children ages 5 and under?							
		Never		Seldom	Sometimes	Often	Very often
1	The clinic coordinates care for patients requiring complex dental treatment (for example, pulpotomies, crowns, and space maintenance)	( )		( )	( )	( )	( )
2	The clinic coordinates care for patients requiring complex medical treatment (for example, cystic fibrosis, sickle cell disease, congenital heart defect, leukemia, and transplants)	( )		( )	( )	( )	( )
3	The clinic coordinates care with the Medical Officers	( )		( )	( )	( )	( )
4	The clinic coordinates care with social services, schools, and care facilities	( )		( )	( )	( )	( )
Which of the following applies to your oral health care practice for children ages 5 and under?							
		Never		Seldom	Sometimes	Often	Very often
1	The clinic provides recall visits and continuing care over the long term	( )		( )	( )	( )	( )
2	Dental Officers and Dental Therapists schedule patients for recall (for example, three months, six months, and one year) based on caries risk and other individual factors	( )		( )	( )	( )	( )
3	The clinic participates in community programs (for example, oral disease prevention, screening, and referral)	( )		( )	( )	( )	( )
Part C: Awareness of the Dental Home Concept							
Have you ever heard of the “dental home”?			( ) Yes ( ) No				
The dental home is described by the ongoing relationship between:			( ) I don’t know ( ) A dentist and the patient ( ) Any medical or dental professional and the patient				
By when should the dental home be established?			( ) I don’t know ( ) No later than 1 year of age ( ) No later than 3 years of age				
